# A Critical Review of Psychological Safety in Psychoeducation for Patients with Anxiety Disorders and Implications for Medical Education in Psychiatry

**DOI:** 10.1192/bjo.2025.10313

**Published:** 2025-06-20

**Authors:** Chao Tian Tang, Palanivelu Sendhil Kumar, Ho Teck Tan, Siew Fai Liew, Shian Ming Tan

**Affiliations:** Sengkang General Hospital, Singapore, Singapore

## Abstract

**Aims:** Psychoeducation for patients with anxiety disorders has been associated with reduced symptoms, improved quality of life, and increased response to treatment. However, there is much heterogeneity in such interventions. Patients with anxiety disorders can have maladaptive cognitive patterns such as catastrophic thinking, attentional biases toward threat, and cognitive avoidance. These patterns can interfere with the processing and assimilation of psychoeducational content, potentially reducing its effectiveness. Our hypothesis is that psychological safety plays a key role in increasing the effectiveness of psychoeducational interventions for patients with anxiety disorders. We aim to critically review the role of psychological safety in such interventions and to explore its impact on medical education in psychiatry.

**Methods:** Sources were identified through searches via databases including PubMed, PsycINFO, Scopus, Cochrane Library, and Google Scholar. Results were then critically analysed with key themes extracted to evaluate the role of psychological safety in psychoeducation. A narrative synthesis was then performed, exploring the influence of this on medical education in psychiatry.

**Results:** Several key themes were identified. Psychological safety has a mediating role between the quality of doctor-patient communication and patients’ self-disclosure, which can be limited by various fears, including a fear of negative judgment in this population. Patient engagement, which has a multidimensional construct, is also impacted by the presence of psychological safety which increases patient openness and comfort. Psychological safety also aids collaborative efforts within the healthcare ecosystem, positively impacting the outcomes of psychoeducational processes. The influence of psychological safety on the concept of the therapeutic interpersonal relationship in psychoeducation was also explored, with implications for open communication and the perception of threats. Proposed enhancements to assessments and curriculum for educational efforts in anxiety disorders and corresponding psychoeducational interventions through the active provision of psychological safety concepts were discussed.

**Conclusion:** This critical review highlights the pivotal role of psychological safety in enhancing the effectiveness of psychoeducational interventions for patients with anxiety disorders. By equipping clinicians to create safe environments, these efforts can optimize psychoeducational interventions and ultimately improve care for patients with anxiety disorders. Targeted studies on specific subgroups of patients with anxiety disorders should be performed to better qualify and quantify the impact of psychological safety in psychoeducational interventions in these subgroups.

